# Case report: Characterization and bioinformatics analysis of non-O1/O139 *Vibrio cholerae* strain isolated from a choledochoduodenal fistula patient with septicemia

**DOI:** 10.3389/fmed.2024.1458143

**Published:** 2024-10-03

**Authors:** Wei Yang, Peibo Li, Songping Lei, Yaqing Yu, Shuangjuan Liu, Chengdong You

**Affiliations:** ^1^Department of Infectious Diseases, People’s Hospital of Xiushan County, Xiushan, China; ^2^Department of Tuberculosis Diseases, Chongqing Public Health Medical Center, Chongqing, China; ^3^Centers for Disease Control and Prevention of Xiushan County, Xiushan, China; ^4^Department of Clinical Laboratory, People’s Hospital of Xiushan County, Xiushan, China

**Keywords:** *Vibrio cholerae*, bioinformatics analysis, choledochoduodenal fistula, virulence factor, septicemia

## Abstract

The gram-negative bacterium *Vibrio cholerae* (VC) is divided into multiple serogroups, with groups O1 and O139 responsible for cholera. Conversely, *Vibrio cholerae* belonging to the non-O1/non-O139 group (NOVC) does not produce cholera-causing toxins. Insufficient understanding of the frequency of NOVC causes fear during the early detection phase. Acute gastroenteritis is often caused by NOVC, while extra gastrointestinal infections are less common. In the case described here, the patient had a postoperative choledochoduodenal fistula due to prior choledochotomy. In August 2023, he was hospitalized with fever and diarrhea. The gram-negative bacilli *Vibrio cholerae* was isolated from a blood specimen using matrix-assisted laser desorption/ionization time-of-flight mass spectrometry (MALDI-TOF MS). The strain was identified as non-O1/O139 by serum agglutination tests. Subsequent whole-genome sequencing and database analysis revealed that the strain possessed resistance genes such as CRP, varG, almG, and QnrVC4, as well as various virulence factors such as RTX, hlyA, VAS, and T3SS. The phylogenetic tree analysis indicated that CQ23-0008VC had close relationship with *cholerae* strains isolated from aquatic environments. The patient was treated promptly and discharged after being admitted with severe symptoms. However, Bioinformatics analysis indicated that the virulence factors that were identified in the bacteria were significant; thus, these virulence factors can indicate to medical professionals that a patient could have a septicemia caused by NOVC.

## 1 Introduction

The severe infectious diarrheal illness known as cholera is caused by the O1 and O139 strains of *Vibrio cholerae* (1). Although NOVC primarily leads to intestinal infections, it can also result in parenteral infections, particularly septicemias, in individuals with compromised immune systems. *Vibrio cholerae* can induce diarrhea resembling cholera. Although the exact etiology is unknown, several case reports indicate that individuals with neoplasia, diabetes mellitus, and chronic liver disease have higher rates of NOVC septicemias (2–5). Thus, highlighting the high fatality rate associated with these parenteral septicemias is crucial, as they frequently lead to severe complications such as infectious shock and multiorgan failure (6).

The etiology of NOVC remains unknown; however, virulence gene analysis in published cases revealed significant virulence factors, including gene clusters for hlyA, toxR, hapA, rtxA/rtxC, nanH, zot, stn, ompU, luxS, the type VI secretion system (T6SS), and the type III secretion system (T3SS) (7–9). Variations in the distribution of these virulence factors are directly linked to the pathogenicity of NOVC and its various clinical manifestations (10). In addition, sepsis is relatively rare in patients with Choledochoduodenal fistula. The present study describes the whole sequence of an isolate of NOVC from a choledochoduodenal fistula patient with septicemia.

## 2 Materials and methods

### 2.1 Strain isolation and identification

Blood samples from a patient with a choledochoduodenal fistula were collected for strain identification. The strain was subjected to gram staining and examined under a microscope. Subsequently, the strain was transferred to a blood plate and incubated for 16 h. Individual colonies were isolated for serological and biochemical identification.

### 2.2 Antibiotic susceptibility test

The drug susceptibility test was performed using the Kirby-Bauer (K-B) method recommended by the National Clinical Laboratory Procedures (China). The 2016 edition of the American Committee for Standardization of Clinical Laboratories’ Drug Susceptibility Test Standards was used to evaluate the results (11). The findings indicated sensitivity to the following drugs: levofloxacin, tetracycline, cefoxitin, cefuroxime, imipenem, meropenem, ampicillin, ceftazidime, cefotaxime, cefepime and cotrimoxazole.

### 2.3 Whole-genome sequencing and gene function analysis

Sequencing was performed using the MGISEQ200RS (Beijing Genomics Institute, China) platform with Illumina PE100 technology for bipartite sequencing. The MGAP (Beijing Genomics Institute, China) platform was used for sequence assembly and splicing. Average nucleotide identity (ANI) analysis and strain identification were conducted on the assembled sequences using the pubMLST database (https://pubmlst.org/species-id) and the ANI calculator (Environmental Microbial Genomics Laboratory gatech.edu). Gene sequences were compared to the KEGG, COG, CAZyme, VFDB, and CARD databases using Prokka software (version 1.14.6) to obtain genome annotation information. The final assembled complete genome was uploaded to the NCBI database (www.ncbi.nlm.nih.gov) under accession number JBCQWS000000000.

### 2.4 Phylogenetic tree construction

To understand the possible origin of CQ23-0008VC bacteria about the genetic evolution of different *vibrio*, a phylogenetic tree was constructed through the Mega software (version 11) according to the. *V. cholerae* O1 biovar EI Tor str. L-3226 (access number: GCA_000600255.1) served as the reference strain, the genome of *Vibrio* was retrieved from the NCBI database (https://www.ncbi.nlm. nih.gov).

## 3 Results

### 3.1 Case presentation

**Figure 1:**
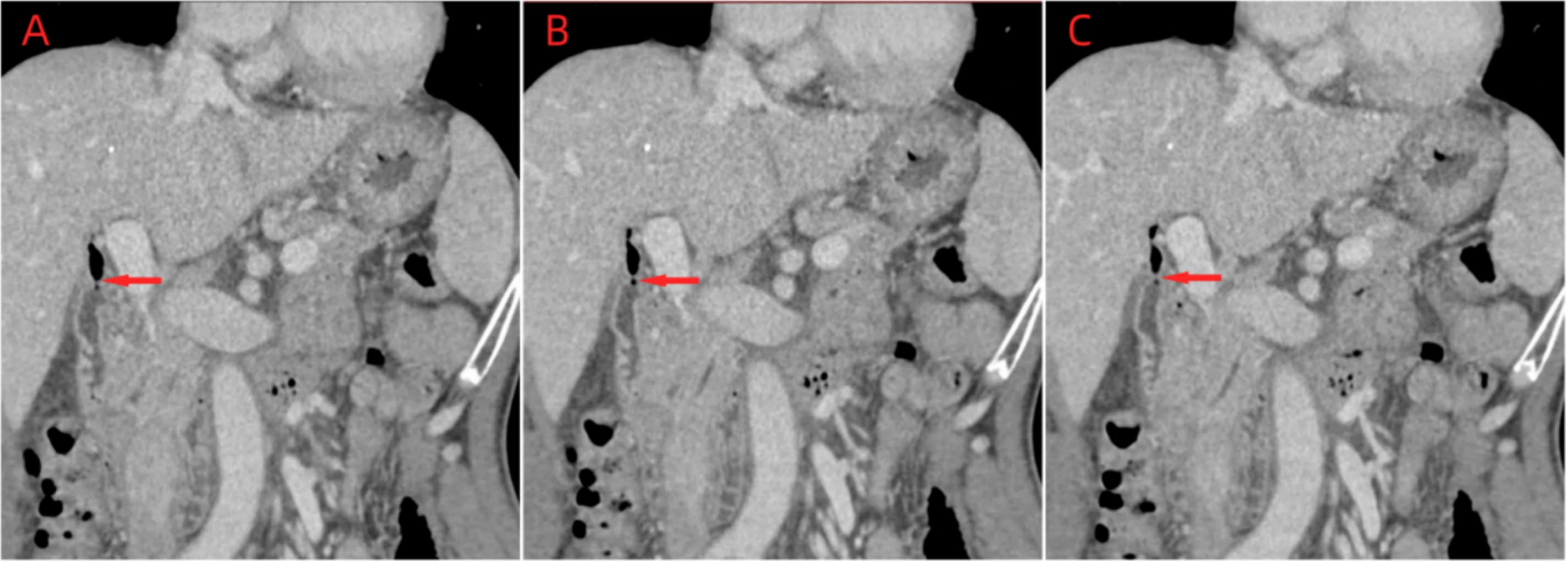
Abdomen enhancement CT imaging of choledochoduodenal fistula.

In August 2023, a 64-year-old male patient who experienced chills, fever, nausea, vomiting, diarrhea, and altered stomach contents. He had undergone a cholecystectomy 30 years prior, suffered from long-term sporadic vomiting, and maintained a regular diet. Enhanced computed tomography (CT) of the abdomen revealed Choledochoduodenal fistula (Figure 1). On the night of admission, the patient presents with chills and fever. A blood culture was obtained, the patient was treated with ceftazidime (2 g every 8 h) for anti-infection. On August 7, blood culture revealed the growth of Gram negative bacteria, which were identified as *Vibrio cholerae* and transferred to blood agar plates. The strain was identified as *V. cholerae* of the non-O1/non-O139 group, which did not produce the virulence factors ctxAB and tcpA. On August 8, the strain was sent to the Center for Disease Control and Prevention (CDC) of Chongqing for gene sequencing. Following an 8-day course of ceftazidime anti-infection therapy, the patient was cured and discharged from the hospital. Prior to illness, the patient had no contact with other patients with diarrhea or with sea or river water. The main clinical features and laboratory tests during the hospitalization of this patient are presented in Table 1.

**Table 1:** The clinical features of the patient during the hospitalization.

Items	Results	Items	Results
T (°C)	39.2	TBIL (umol/L)	61.8
WBC (10^9^/L)	12.3	HBV-DNA (IU/ml)	2.34 × 10^2^
NEU (%)	87.5	Abdominal CT	Choledochoduodenal fistula
PCT (ng/ml)	13.167	Basic disease	Hepatitis B virus carriers
ALT (U/L)	145	Past medical history	Cholecystectomy, long-term sporadic vomiting

### 3.2 Strain isolation and identification

**Figure 2:**
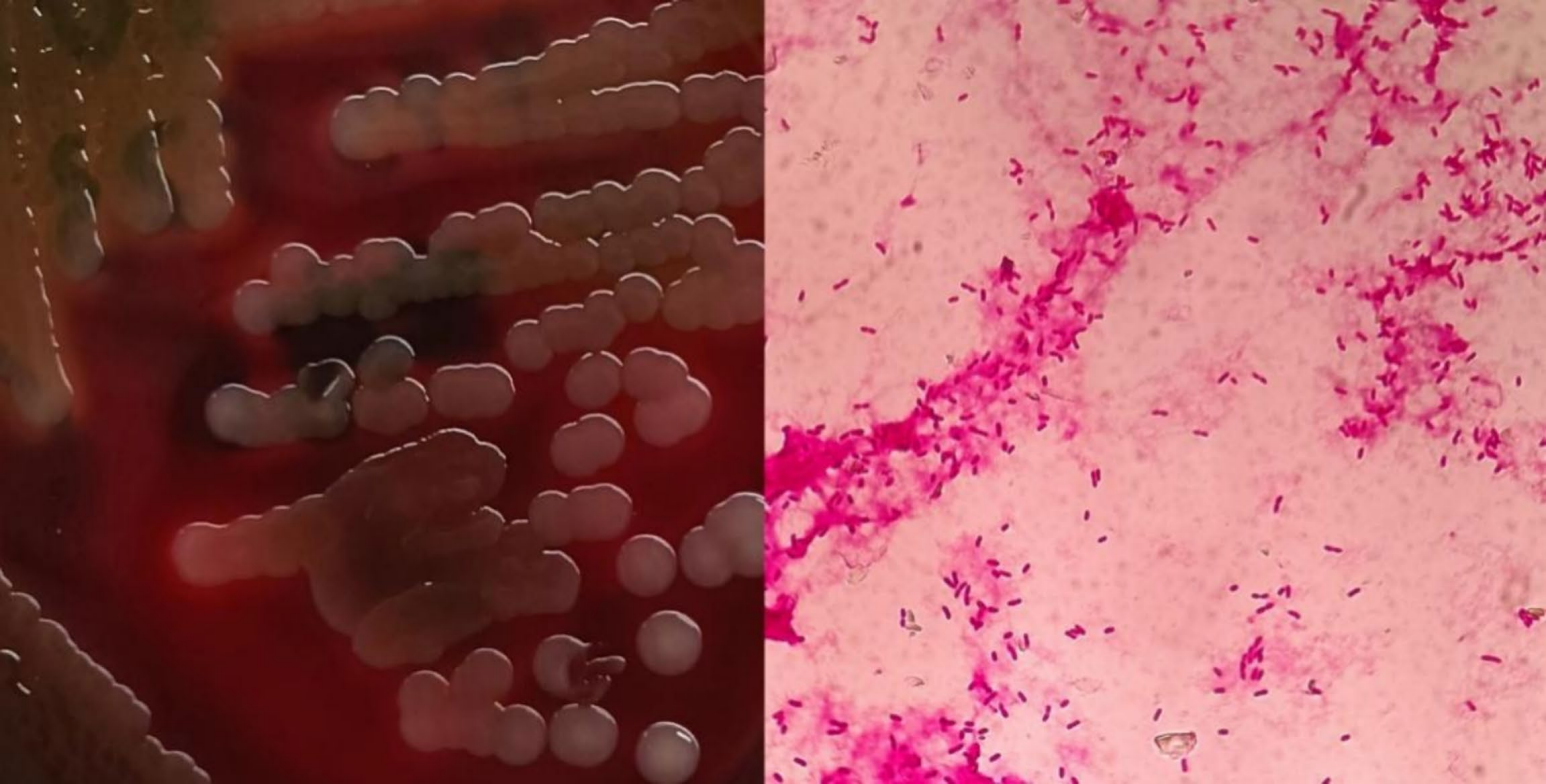
Blood agar plate and morphological characteristics were observed under microscopy after gram staining.

After sixteen hours, blood cultures were positive for gram-negative bacteria upon microscopic examination (Figure 2). Within a day, *β*-hemolytic colonies appeared on the transgenic blood agar plate. MALDI-TOF MS confirmed the identification of this strain as *V. cholerae*. The local CDC identified the bacterium as a non-O1, non-O139 *V. cholerae* strain, designated CQ23-0008VC, after O1 and O139 serum agglutination tests returned negative results.

### 3.3 Genome assembly and genome annotation information analysis

**Figure 3:**
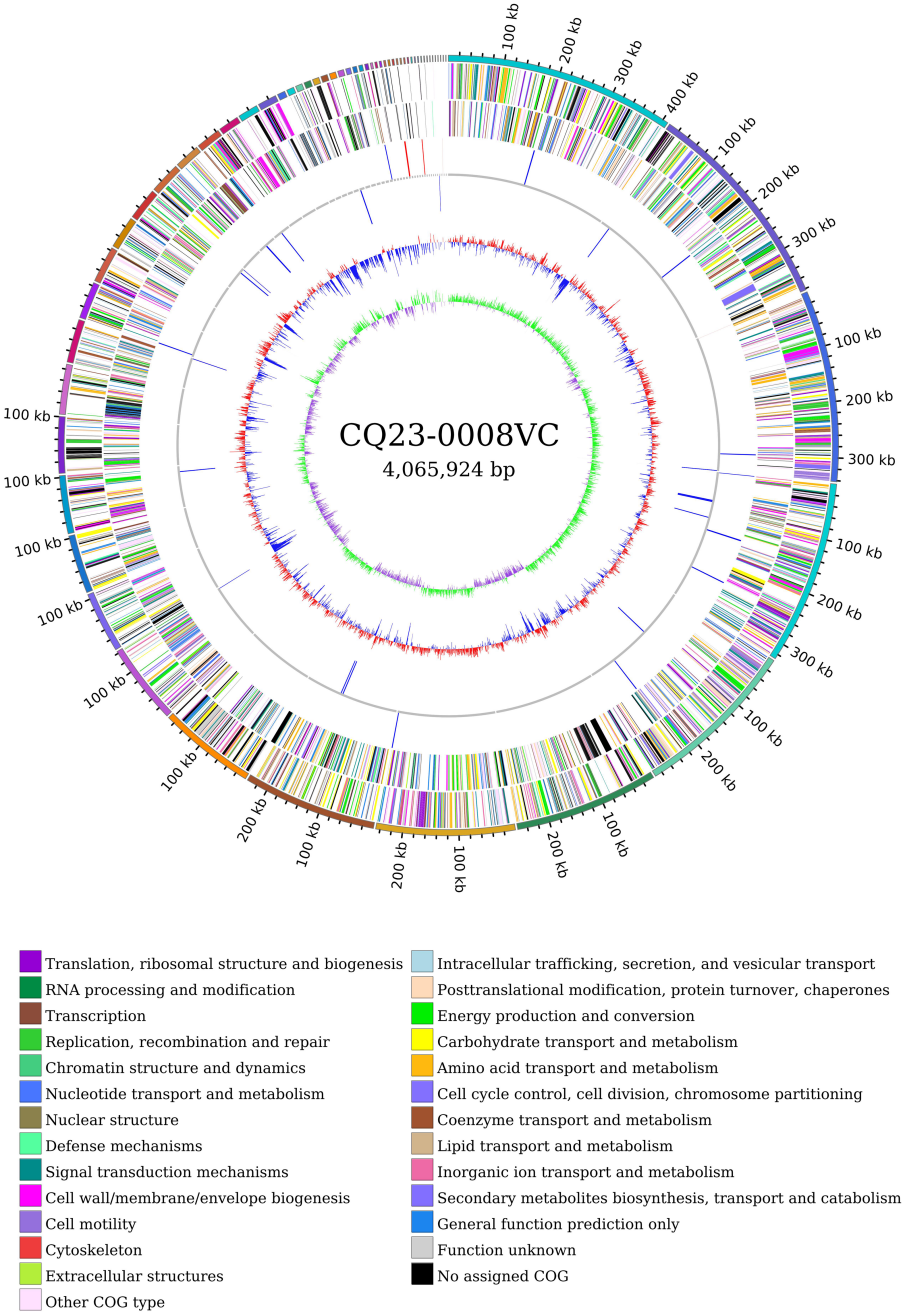
Genome circle map of CQ23-0008VC sequence.

Following sequencing, the original CQ23-0008VC sequence was subjected to quality control, assessment, assembly, and circular genome mapping using Circos software (Figure 3). The CQ23-0008VC genome was estimated to comprise 4,065,924 bp, encoding 3,664 genes with a 47.48% GC content. The genome included 76 tRNAs, 7 rRNAs, and 1 tmRNA among the noncoding RNAs. The sequences were posted to the PubMLST website’s rMLST database, where they matched 100% to *V. cholerae* ST680. The identification results revealed alleles encoding adk 2, gyrB 23, mdh 15, metE 9, pntA 59, purM 1, and pyrC 35. Using the ANI calculator, 98.37% of the sequences were identified as ANIbs. A total of 3,580 (97. 71%) genes were annotated through comparison with other databases: 2,814 genes were annotated by the COG database, 2,323 genes by the KEGG database, and 186 genes by the VFDB database.

**Figure 4:**
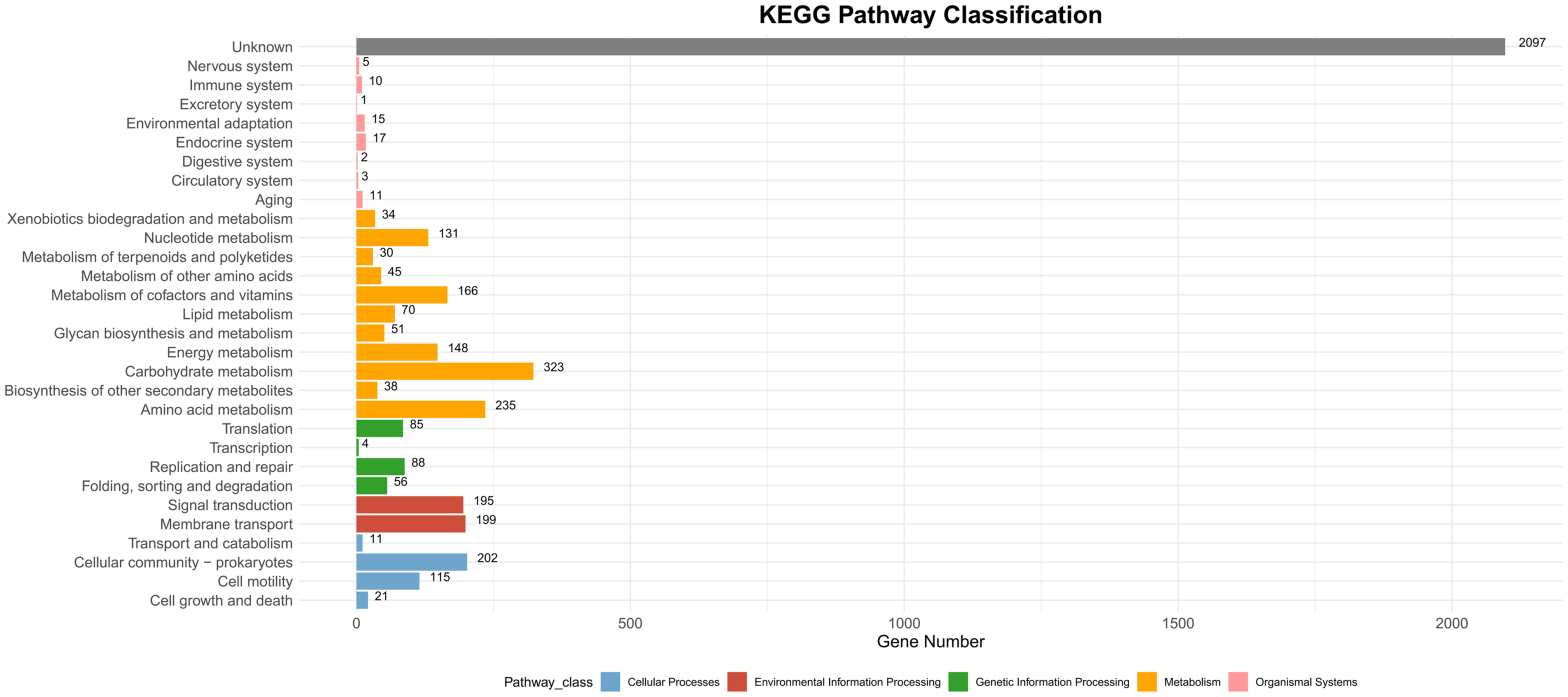
Distribution of KEGG functional annotations of *Vibrio cholerae* CQ23-0008VC.

**Figure 5:**
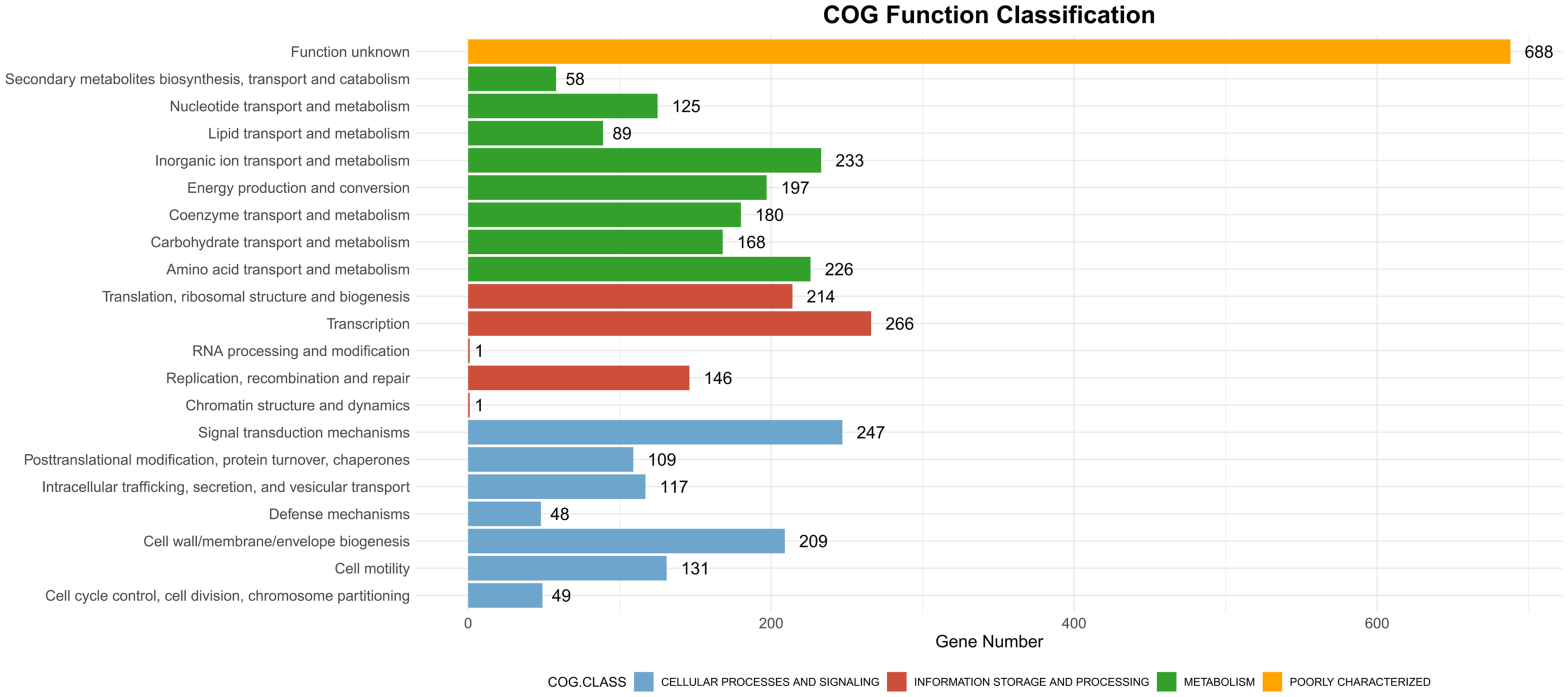
Distribution of COG functional annotations of *Vibrio cholerae* CQ23-0008VC.

CAZyme database analysis was performed to identify six major categories of carbohydrate-active enzymes in the CQ23-0008VC genome, suggesting that the bacterium had diverse functional capabilities and adaptability. The CQ23-0008VC genome contained 2,323 genes linked to metabolic pathways, which were identified by analysis via the KEGG database. These genes were classified into four categories: metabolism, genetic information processing, cellular motility, and organic systems (Figure 4). Genes were classified into three main functional types and assigned to twenty categories in the COG database. These categories included metabolism, information processing and storage, cellular activities, and signaling. The metabolic pathways of 688 genes remain unknown (Figure 5). Using the VFDB database, genes in the CQ23-0008VC genome that may encode virulence factors were investigated. The genome contained 160 other virulence-related factors, such as rtxA and hlyA, but lacked the CTX virulence factor (Table 2).

**Table 2:** Prediction of virulence factor genes of the *Vibrio cholerae* Strain CQ23-0008VC.

Functional description	Virulence factor	Gene name
Adherence	Toxin-coregulated pilus (type IVB pilus) Type IV pilus	MshB-mshN PilA-pilD
Antiphagocytosis	Capsular polysaccharide	CpsA-cpsD, cpsF, rmlD, wbfB wbfU, wbfV/wcvB, wbfY, wbjD/wecB wecA, wecC, wza, wzb, wzc
Chemotaxis and motility	Flagella	cheA, cheB, cheR, cheV, cheW, cheY, cheZ filM flaA-flaE, flag, flaI flgA-flgN flhA, flhB, flhF, flhG fliA, fliD-fliS flrA, flrB, flrC motA, motB, motX, motY
Enzyme	Metalloproteinase Neuraminidase	hap/vvp nanH
Iron uptake	Enterobactin receptors Heme receptors Periplasmic binding protein-dependent ABC transport systems Vibriobactin	irgA, vctA hasR, hutA, hutR vctC, vctD, vctG, vctP viuC, viuD, viuG, viuP vibA-vibF, vibH viuA, viuB
Quorum sensing	Autoinducer-2 Cholerae autoinducer-1	luxS cqsA
Secretion system	T3SS2 secreted effectors T3SS2 VAS effector proteins VAS type VI secretion system	vopL vcrD2, vscC2, vscN2 hcp-2, vgrG-1, vgrG-2 vasA-vasK
Toxin	RTX toxin Thermolabile hemolysin *Vibrio cholerae* cytolysin	rtxA, rtxB, rtxC, rtxD tlh hlyA
Serum resistance	LPS rfb locus (Klebsiella)	–

### 3.4 Resistance phenotypes and resistance genes

The strain was found to be sensitive to ampicillin, methotrexate/sulfamethoxazole, cefoxitin, cefuroxime, imipenem, tetracycline, levofloxacin, cefepime, cefotaxime, meropenem, and ceftazidime (Table 3). The CQ23-0008VC genome harbored four resistance genes: varG, almG, QnrVC4, and CRP (Table 4).

**Table 3:** Antimicrobial resistance profile of the *Vibrio cholerae* Strain CQ23-0008VC.

Antimicrobial agent	KB (mm)	KB/MIC breakpoint	Result
Levofloxacin	25	≤13 ≥ 17	S
Cefepime	34	≤18 ≥ 25	S
Cefotaxime	36	—	S
Meropenem	27	≤19 ≥ 23	S
Ceftazidime	28	≤17 ≥ 21	S
Ampicillin	21	≤13 ≥ 17	S
Methoxybenzyl/Sulfamethoxazole	30	≤10 ≥ 16	S
Cefoxitin	22	≤14 ≥ 18	S
Cefuroxime	23	≤14 ≥ 18	S
Imipenem	24	≤19 ≥ 23	S
Tetracycline	30	≤11 ≥ 15	S

**Table 4:** The antibiotic resistance genes of the *Vibrio cholerae* Strain CQ23-0008VC annotated in CARD.

Gene name	Categories
CRP	Macrolide antibiotic, fluoroquinolone antibiotic, penam
VarG	Cefazolin antibiotic
almG	Peptide antibiotic
QnrVC4	Fluoroquinolone antibiotic

### 3.5 Phylogenetic tree analysis of *Vibrio cholerae* CQ23-0008VC

**Figure 6:**
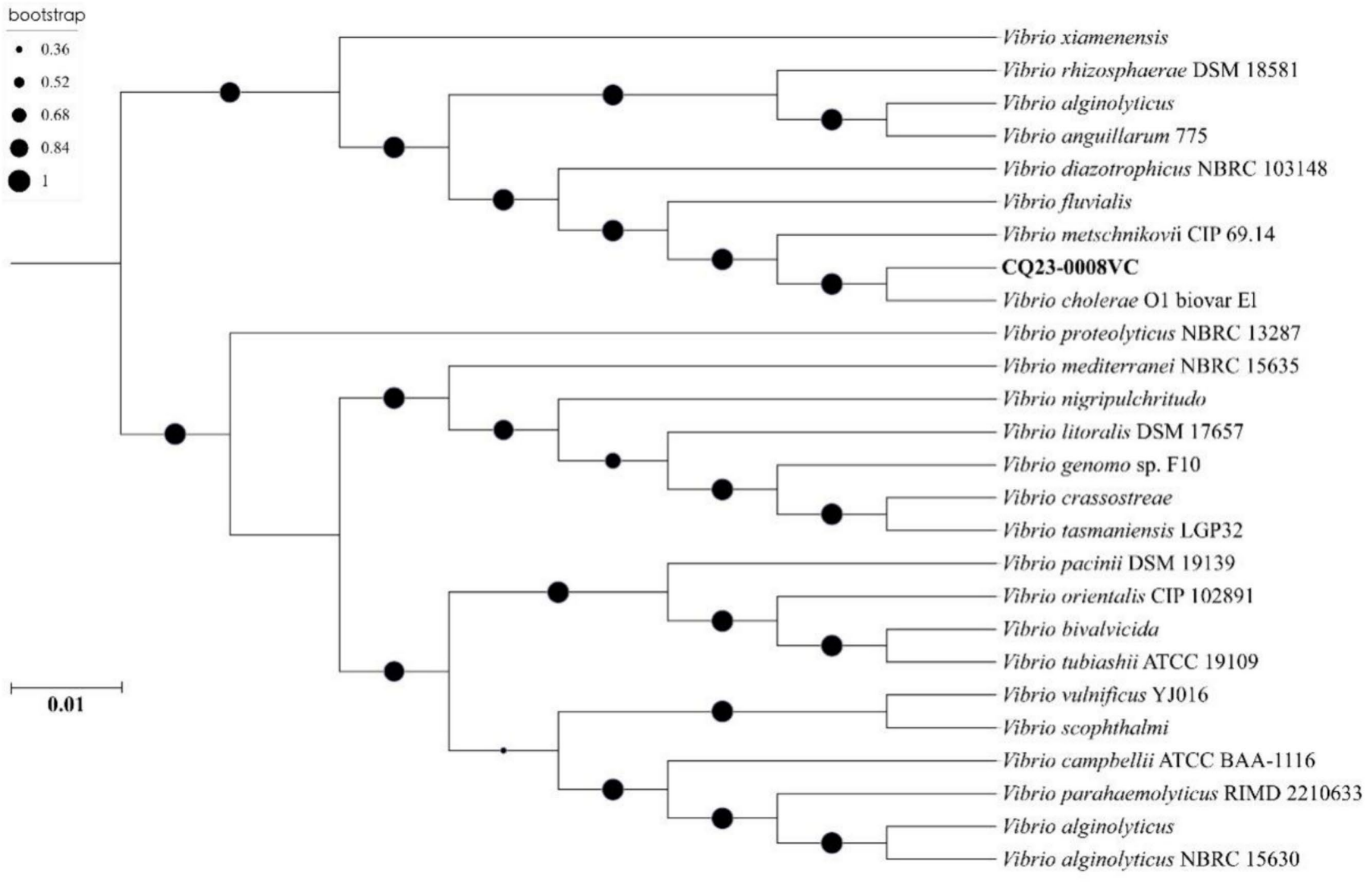
A nucleotide sequences-based phylogenetic tree of 25 *Vibrio cholerae* strains.

Phylogenetic tree was constructed using nucleotide sequences of these 25 isolates strains from GenBank. The results indicated that CQ23-0008VC was the closest in genetic relationship to a *V. cholerae* strain O1 biovar EI. Besides, CQ23-0008VC belonged to the same genetic evolutionary lineage as Vibrio, which originates from marine, soil, and river environments (Figure 6).

## 4 Discussion

Clinicians have started to take NOVC more seriously due to documented cases of extraintestinal infections, despite its typical reputation as a weakly pathogenic or asymptomatic colonizer (12). These infections increasingly include skin infections, meningitis, septicemias, lung infections, and plasma infections. In some instances, these infections can escalate into infectious shock and potentially fatal multiorgan failure (13–16). Given the relatively large hepatopathic population in China, paying sufficient attention to NOVC is crucial as it is more likely to infect immunocompromised patients with chronic liver disease, diabetes mellitus, malignant tumors, and other conditions, often resulting in serious septicemias (17–19).

In 2018, a retrospective analysis of three disseminated cases of NOVC bacteremia by Chinese scholars found that extraintestinal infections of nontoxigenic non-O1/ non-O139 *V. cholerae* are highlighted in hepatitis B cirrhosis patients (7). Multiple literature reviews have also confirmed that liver diseases, such as hepatic cirrhosis, liver cancer are a risk factor for NOVC infection (10, 20, 21). In this case study, the patient had a choledochoduodenal fistula, which could be the pathological pathway of NOVC invasion, and was also an HBsAg carrier, but the patient did not know that he carried hepatitis B virus before. In addition, this patient had been diagnosed with NOVC infection in August, which is consistent with the time reported in the literature reviews (10). Epidemiological analysis revealed that the patient’s history of pathogenic exposure was unclear. In several instances, there was no obvious source of exposure, and NOVC was not found in other family members or water supplies.

Comparison of the strain’s sequencing data to the KEGG, COG, and CAZyme databases indicated that the strain’s growth mode and metabolic pathways were similar to those of other NOVCs (7, 22). Although the patient’s diarrhea symptoms were not as severe as those caused by cholera, strain CQ23-0008VC carried more virulence factors than did other strains in the VFDB database. This finding could be related to the stimulation of other virulence factors or to dysbiosis of the intestinal flora resulting from septicemia. The clinical presentation and virulence factors reported in this case align more closely with those observed in previous instances of septicemia caused by NOVC (23, 24).

The drug sensitivity results demonstrated that the CQ23-0008VC strain was sensitive to other antimicrobial agents, including quinolones, cephalosporins, and sulfonamides. This strain did not develop resistance to ampicillin or imipenem, which may be related to its origin. Bacterial resistance is closely linked to patient prognosis. According to the CARD database, the resistance genes detected in strain CQ23-0008VC were CRP, varG, almG, and QnrVC4, There were fewer resistance genes in this strain than those found in other reported NOVC strains, which may explain its sensitivity to most antimicrobial agents and suggests variability in resistance rates among different NOVC strains. This result was further supported by a meta-analysis (25). Despite being susceptible to antibiotics such as quinolone, penicillin, aminoglycoside, and macrolides, strain CQ23-0008VC possessed related resistance genes. This phenomenon may be related to the expression levels of resistance genes, their silencing or inactivation, and the genetic backgrounds of the bacteria (26). Therefore, clinicians should consider their assessments on the phenotype characteristics of the strains’ in vito antimicrobial susceptibility, rather than relying solely on evaluations from databases (27, 28). Conversely, when resistance genes are detected in the genome but the isolates show susceptibility in phenotype tests, a critical approach should be taken to interpret the antimicrobial spectrum accurately. Therefore, *in vitro* drug sensitivity testing should be combined with resistance gene monitoring to choose an antibiotic regimen more precisely and prevent the development of drug-resistant bacteria.

Hemolysin A (HlyA), which is extensively distributed in *Vibrio cholerae* and has a distinctive structural domain that attaches to target cells, may significantly contribute to the ability of NOVC to infiltrate the bloodstream (29, 30). The distributions of the T3SS and the T6SS differed between strains; comparison with the VFDB database revealed that the strain in this case had a T3SS rather than a T6SS. Determining the difference in bacterial pathogenicity caused by a T3SS or a T6SS will require further investigation. The T3SS/T6SS might increase the virulence of NOVC strains lacking CT and TCP genes, potentially explaining the severity of some septicemias (31–33). Numerous virulence factors work together during parenteral infection with NOVC, thereby necessitating further in-depth molecular biology research to fully comprehend the pathogenic mechanisms involved in parenteral infection caused by NOVC.

## 5 Conclusion

This study shows that NOVC carries multiple virulence factors and resistance genes and is potentially pathological in humans. Although there is an inconsistency between drug-resistant phenotypes and drug-resistant genes, the monitoring of NOVC resistance genes is helpful for the rational use of antimicrobial drugs. Although NOVC infection is rarely, clinicians should be aware of the possibility of infection with this pathogen in patients with hepatic and biliary tract diseases. Therefore, parenteral infections caused by NOVC should be taken more seriously.

## Data Availability

The datasets presented in this study can be found in online repositories. The names of the repository/repositories and accession number(s) can be found below: https://www.ncbi.nlm.nih.gov/genbank/, JBCQWS000000000.
